# Work practices and the provision of mental-health care on the verge of reform: a national survey of Israeli psychiatrists and psychologists

**DOI:** 10.1186/2045-4015-3-25

**Published:** 2014-07-22

**Authors:** Nurit Nirel, Hadar Samuel

**Affiliations:** 1 Myers-JDC-Brookdale Institute, Smokler Center for Health Policy Research, JDC Hill, POB 3886, Jerusalem 91037, Israel

**Keywords:** Reform, Mental health, Psychologists, Psychiatrists, Managed care, Israel

## Abstract

**Background:**

The State of Israel is preparing to transfer legal responsibility for mental- health care from the government to the country’s four competing, nonprofit health-plans. A prominent feature of this reform is the introduction of managed care into the mental-health system. This change will likely affect the service delivery patterns and care practices of professional caregivers in mental-health services. The study examines psychiatrists’ and psychologists’ patterns of service delivery and practice, and their attitudes toward the reform’s expected effects, focusing on the following questions: To what extent do today’s patterns of service delivery suit a managed-care environment? To what extent do professionals expect the reform to change their work? And do psychiatrists and psychologists differ on these questions?

**Methods:**

A survey of 1,030 psychiatrists and psychologists using a closed mail questionnaire for self-completion was conducted from December 2011 to May 2012.

**Results:**

Substantial differences were found between psychiatrists’ and psychologists’ personal and professional characteristics, work patterns, and treatment-provision characteristics. In addition, the study identified gaps between the treatment-provision characteristics of some of the professionals, mostly psychologists, and the demands of a managed-care environment. Moreover, a high percentage of the mental-health professionals (mostly psychologists) do not expect improvement in the quality of care or its accessibility and availability following the reform. However, those reporting practices associated with managed care (e.g. short-term treatment, compliance with monitoring procedures, and emphasis on evidence-based treatment) are less likely to expect negative changes in the provision and quality of care after the reform.

**Conclusions:**

Steps need to be taken to reduce the gaps between the treatment-provision characteristics of the professionals and the demands of a managed-care environment, and there are several possible ways to do so. In order to recruit experienced, skilled professionals, the health plans should consider enabling various work models and offering training focused on the demands of working in a managed-care environment. It is advisable to implement this kind of training also during the training and specialization process by including these topics in the professional curricula.

## Background

Israel’s mental-health system is preparing for major reform, which will establish a legal entitlement of patients to mental health care and transfer responsibility for service provision from the state to health plans (the Mental Health Insurance Reform).

The legislation of the National Health Insurance Law (NHIL) in Israel in 1995 ensured universal access to an extensive basket of benefits. All residents are entitled to join one of four competitive, not-for-profit health plans. However, the mental-health and general health systems have functioned separately as reflected by funding, planning, organization and operating frameworks. The state is both responsible for mental-health services and their major provider: it runs about half of the state psychiatric hospitals as well as the largest network of community mental-health centers that also play a key role in training professionals for mental health. In addition, a large number of self-employed practitioners provide community-based mental-health services, mainly on a private basis. Over the years, the system has come in for a good deal of criticism, namely: the insufficient connection between mental and physical health, the long waiting lists, unmet needs in moderate mental-health conditions, the lack of clear, legally enforceable rights to mental-health services, and the stigma accompanying mental illness and treatment. Apparently, this state of affairs contributed to the high rate of treatment of patients with “soft” psychiatry were offered privately rather than through the public clinics [1,2]. According to the NHIL, the responsibility for the provision of mental-health services was to be transferred from the state to the health plans within three years of the legislation. The health plans and Ministry of Health (MoH) began mobilizing for the change as operative dates were set – about five times. Yet the attempts to implement the reform failed and responsibility was never transferred. Nonetheless, all the health plans did and do provide insurees with an ambulatory mental-health service alongside existing services in state ambulatory frameworks. The provisions were partially developed in the course of the health plans’ mobilization whenever reform seemed imminent, and were neither expanded nor cancelled with every delay. A health plan may offer the service as part of its basic basket or as part of its supplementary-insurance services. All the health plans offer it as a limited service, treatment is provided against a considerable co-payment charge and the health plans are not committed to accessibility and availability. Moreover, the changes have hardly mitigated the abovementioned criticism of the mental-health system [3].

Ultimately, in April 2012, after 17 years of deliberations, the government issued a directive that the state mental-health services be transferred to the basket of services under the responsibility of the health plans [4]. At the time of writing, the mental-health system finds itself in a three-year process to execute the insurance reform. During this period, decisions will be made about the way that the health plans are to organize and deliver services.

The main goals of the reform are to improve quality of care, to expand availability and accessibility of services (especially to underserved populations), and to increase efficiency. To a large extent, the expectation of improved quality stems from three main components: the introduction of elements of managed care into the mental-health system, integrated mental and physical treatment, and increased funding for mental health. The reform also raised concerns regarding the adequacy of funding levels, possible medicalization of mental-health, and the erosion of professional relations between service systems.

The literature on the provision of mental-health services in an era of managed care notes that the main aims of the latter are to curb costs while ensuring quality of care through regulation and monitoring of the treatment process and outcomes [5,6].

In the US, implementing managed care has led to new patterns of service provision. Many professionals gave up solo practices in favor of group practices, generally using a one-stop shop model where a group of professionals offer treatment at the same place, each in their own field. Since managed-care organizations usually limit the number of therapy sessions and favor short-term treatment, therapists had to increase the number of patients and decrease the time devoted to each to maintain their income levels. Under managed care, many psychiatrists practice less psychotherapy and write more prescriptions, while many psychotherapists adopt short-term treatments focusing on improving patient functioning and reducing symptoms [6-11].

Other characteristics of managed care are monitoring and controlling the therapeutic process. Almost all service providers under managed care are obliged to work according to clinical guidelines, presumably to establish standardization and an empirical basis for assessment and treatment. Some argue, however, that such guidelines defy the very nature of psychotherapy [12], which is too complex to lend itself to standard intervention [13]. Furthermore, studies have shown that most psychologists do not believe that managed-care organizations will safeguard patient confidentiality while monitoring the care provided, and that this failing will harm patient-therapist relationships [7,13].

Many psychologists also believe that working under managed care reduces the use of psychological tests – especially time-consuming ones – in favor of brief self-reporting and accounts [14]. Indeed, the selection and number of tests, and the time devoted to them, have decreased in frameworks of managed care [15].

Another impact of managed care relates to inter-professional competitiveness. It was found that many Managed Behavioral Health Organizations (MBHOs) in the US replaced psychiatrists with different, less expensive therapists, such as psychologists and social workers [16]. An examination of the employment market of managed care in mental health showed a drop in demand for psychologists and a rise in demand for other professionals [6]. In the 1990s in the US, the income gap between psychologists and clinical social workers narrowed [17]. Moreover, the demand for psychology services appears to have dropped. In the wake of this, some have predicted an over-supply of psychologists and recommended controlling the employment market by limiting professional training [18].

Note that the impact on mental-health services in managed care in the US is different from that envisaged in Israel’s reform. Since Israel has universal insurance, the entire population will be eligible for care. Consequently, the number of patients may increase as will the demand for mental-health professionals. In addition, the main goal of the MBHOs in the US is to reduce the costs of care [17]. In Israel, on the other hand, the transfer of responsibility for mental-health care from the state to the health plans is also aimed at rendering it more available and accessible to the general public. Since Israel’s health plans are all non-profit organizations, it might well be possible to balance quality of care and provision of professional care with efficiency and reduced costs.

The Mental Health Insurance Reform is a significant change for both the professionals working in the field and the health plans. The reform leaves many of the decisions regarding the organization and provision of services to the health plans. These decisions will likely affect the daily work of professional caregivers in mental health.

This exploratory, descriptive study examines the patterns of service delivery and practice by psychiatrists and psychologists, and their views and perceptions of the effects of reform prior to the decision on its implementation. The study aims to furnish policymakers with elaborate data on the mental-health services provided by Israeli psychiatrists and psychologists,on the eve of the reform, and to contribute to its continued planning and the provisions being made by the health plans for its full implementation. It will also supply baseline data for a future study examining the long-term impact of the reform on the issues examined here. It will thus contribute too to the long-term planning and improvement of mental-health services.

The study goal was to examine (on the eve of the reform) the work of Israeli psychiatrists and psychologists from the following aspects: 1) work patterns, service delivery and provision of care (including variables related to working with organizations of managed care); 2) the nature of contact between therapists and primary physicians; 3) the attitudes of professionals towards the reform and their perception of its expected impact on the work patterns and provision of care.

The discussion of the results revolves around the following issues: To what extent do today’s patterns of service delivery suit a managed-care environment? To what extent do professionals expect the reform to change their work? And do psychiatrists and psychologists differ on these questions?

## Methods

### Study design

a. **Preliminary** open **interviews** with 14 psychologists and 16 psychiatrists involved in service delivery and policymaking in the mental-health system. Results from these interviews, held in 2010, were used to construct a questionnaire for the second phase of the study.

b. **Cross-sectional study** – A survey of mental-health professionals in Israel using a closed questionnaire for self-completion was conducted from December 2011 to May 2012 (prior to the government directive to transfer responsibility from the state to the health plans).

### Study population, sampling framework and sample

The study encompassed all specialist psychologists in clinical and medical psychology certified in the Register of Psychologists and Physicians as specialists in psychiatry^a^.

The sampling frameworks were: (a) the roster of psychologists registered at the MoH. In 2010, the roster contained some 2,800 specialist clinical and medical psychologists of working age – up to 65; (b) the MoH roster of medical professions listing specialist physicians in psychiatry. In 2010 the roster contained nearly 1,100 psychiatrists of working age – up to 65. In total, the study population numbered 3,900 psychiatrists and psychologists (N=3,900).

Simple random sampling was employed, of about 40% of psychologists with a specialist certificate in the abovementioned specialties and all psychiatrists of working age; a total of 1,940 sampled individuals.

The groups of psychiatrists and psychologists differed significantly on every background variable: compared with the psychiatrists, the psychologists had a higher rate of women (73% vs. 48%), were slightly younger on average (50 vs. 53), had a higher rate of Israeli-born (76% vs. 39%), a higher rate of Israeli-trained (88% vs. 45%), and a higher rate using Hebrew as their main treatment language (90% vs. 82%). The psychologists also had fewer years of experience (under 20) in the profession (55% vs. 42%).

### Data collection

In keeping with the guidelines of the MoH legal bureau and data committee, subjects were sent a preliminary letter from the body responsible for professional licensing and registration. The letter explained the study goals, noting the researchers’ obligation of confidentiality. Individuals uninterested in completing the questionnaire were asked to state the same by return mail, email or telephone, and were not sent the questionnaires. These guidelines were set to meet the demands of the Database Law and to permit “informed consent” to participating in the study.

It was found that 201 (9.4%) of the psychologists and psychiatrists did not belong to the study population (were not working as mental-health professionals in Israel). Of the 1,940 sampled individuals in the study population, 1,031 completed the questionnaire (n = 1,031) – 582 psychologists and 448 psychiatrists, a response rate of 53.2%: 58% of the former and 48% of the latter); 148 subjects were not located; i.e., they never received the request to participate in the study. If we calculate the response rate only among recipients of the request, we obtain a 58% response rate (62% of the psychologists and 53% of the psychiatrists).

The refusal rate was 12.6%. Of the letter recipients, 6.5% (123 individuals) took advantage of the refusal option by return mail and thus did not receive the questionnaire. A similar rate (122 individuals) refused to participate at a later stage. Less than 1 percent (7 people) did not complete the questionnaire due to language difficulties, and 26% (504 people) did not respond in full during the time allocated for data collection, despite repeated appeals by mail and telephone.

### Study variables

**Demographic variables; background and professional data** – Professional experience (length of time of certification as a professional and specialist), **working arrangement with employer/service supplier** (main and additional) – **form of arrangement with employer/service provider** (employed, self-employed, contractual) in main and additional work^b^, holding a third place of employment, number of years in main place of employment. **Hours of work and full-/ part-time, patterns of service provision** – number of patients, average number of sessions per day, average duration of session, distribution of patients by average duration of therapy; **tools to evaluate effectiveness of treatment** (such as the therapist’s impressions, patients’ self-reporting, reports by other staff members, interviews, structured questionnaires, evidence-based approach to treatment), **budgetary considerations**, **relationship with primary physicians**.

Questionnaire variables relating to **monitoring and control procedures**, and to the **perceived impact** of the reform were examined by measures comprising several items and constructed according to the outcomes of exploratory factor analysis and the number of factors determined by screening tests. Reliability rested on Cronbach’s internal consistency reliability index, alpha (α). Items were measured on a five-point scale ranging from 1 (not at all) to 5 (a very great extent) with a higher score expressing a greater sense of imminent change. Every individual’s score was the average of her/his answers to each item with a high score, from 3.75, signifying a great/very great extent. These were the measures^c^:

**Monitoring and control procedures** were based on the inspection forms for clinical services of the MoH: a 9-item measure (α =0.90) – diagnostic documentation, uniform patient files, writing treatment summaries and recommendations, documenting reasons for termination of treatment, setting treatment goals, documenting treatment plans, updating computerized patient files, documenting outcome measurements, documenting periodic assessments of adherence to treatment plans.

The measures examining the **perceived impact** of the reform were examined through statements relating to expected changes in different aspects of the professionals’ work^b^:

• **Changes in the patterns of work**: A 6-item measure (α=0.75) – Emphasis on budgetary considerations, increased use of forms, paperwork and bureaucracy, monitoring of diagnosis and treatment plans, interference with professional discretion, emphasis on direct contact with patients, limited coordination of services.

• **Changes in the treatment process**: A 5-item measure (α=0.724) – Emphasis on short-term methods, shortening the treatment, more use of medication, less use of psychodynamic tests, less outreach.

• **Changes in the type and number of patient referrals**: A 6-item measure (α=0.76) – More patients of “soft psychiatry,” more patients who currently waive it due to cost, reducing the stigma associated with mental-health treatment, emphasis on early detection and prevention, integration of mental and physical health.

• **Changes in the quality of care**: A 6-item measure (α=0.71) – Unsuitable or insufficient treatment, detriment to severely mentally-ill patients, more referrals to group therapy, employment of unsuitable service providers, improved quality of care.

• **Changes in training and specialization**: A 5-item measure (α=0.9) – Less onsite staff training, fewer hours of consultation and staff meetings, less time devoted to professional updating, seminars, training and conferences, reduction of the range, scope and variety of professional training.

• **Changes in the employment market**: a 4-item measure (α=0.79) – Preference for “less expensive” therapists, competition between various service providers, detriment to workers’ rights, detriment to therapists’ income.

• The respondents were presented with statements related to expected changes in **professional standards and ethics** following the reform. These statements were reduced to a single factor in the analysis. However, since it showed low reliability according to Cronbach’s alpha, no measure was constructed.

• In addition, the extent of **access and availability** of treatment expected after the reform was also examined.

### Statistical analyses

To ensure accuracy of the study estimates, the sample was weighted: Each professional group received a weight according to its relative size in the study population, as follows:

$$\frac{\mathrm{Population size}}{\mathrm{Number of respondents}}=\mathrm{Weight}$$

The study findings were analyzed by cross-tabulation. For some questions, more than one answer was possible. In these cases, the percentages in the tables add up to more than 100%.

The interdependency of categorical variables (measured on a nominal scale) was examined with the Chi-squared test. The significance of the differences between quantitative variables was examined by multivariate analysis (logistic or linear regression), with the T test.

## Results

### Characteristics of work

#### Place and Status of work

The foremost difference in the work characteristics of psychologists and psychiatrists concerns their **main employment**: For over half of the psychologists’ (58%), this was in the private sector whereas for most psychiatrists (some 80%), it was in the public sector. Most psychiatrists (84%) were on salary at their main employment, 11% were self-employed, and some 4% were self-employed by contract with the health plans. Among psychologists, some 40% were on salary at their main employment, 46% worked privately, and 11% were self-employed in their arrangements with the health plans.

Of all the psychologists and psychiatrists, 78% reported having an **additional place of employment**. Here, too, the difference between the two groups was significant: 63% of the psychiatrists worked privately at their other employment. The rate of psychologists working privately at their other employment was 43%, while 29% worked at their other employment on salary, and 12% did so by contract with the health plans. About a third (33%) of the professionals were found to work in more than two employment frameworks. Here, too, the difference was significant: 40% of the psychiatrists worked at a third job compared with 31% of the psychologists^d^.

### Frameworks of care provision

Of the psychologists, 58% worked (in the main place of employment) in their own private offices or in a private institute. In contrast, most psychiatrists (52%) reported working in hospitals, including hospital clinics delivering ambulatory service to community patients and mental-health community clinics located on hospital grounds, (Table 1)^e^.

**Table 1:** Framework of delivery of care in main employment (%)

	**Total**	**Psychiatrists**	**Psychologists**
Main employment**	**100**	**100**	**100**
Hospital psychiatry department	15	35	6
Hospital ambulatory clinic	10	17	7
Mental health clinic/Ministry of Health	9	11	8
Health plan community clinic	5	12	2
Other public institute/facility	8	4	10
Private institute/facility	5	1	7
Private clinic	39	11	51
Other^1^	10	8	11

N (population)=3,900.

n (sample size)=1,030.

**The difference between the groups is significant, p<0.01.

^1^Such as student guidance services, university or college services, National Insurance Institute, areas unrelated to mental health.

### Part-/fulltime employment and working hours

Of the psychiatrists, 81% worked fulltime at their main place of employment compared with 37% of the psychologists. Psychologists worked an average of 35 weekly hours; psychiatrists – an average of 48 hours. There was a significant difference between psychologists according to main place of employment in the public or private sector: 25% of the former worked fulltime compared with 46% of the latter; conversely, 64% of the former worked up to half-time compared with only 36% of the latter. Similarly, public-sector psychologists worked an average of 38 weekly hours; private-sector psychologists – an average of 33.

The professionals were also asked about the number of sessions they have with patients in the course of treatment^f^: 47% of the psychologists said patients received an average of 40 sessions compared with 17% of the psychiatrists’ patients.

Another significant difference was found between psychologists by their main work, in the public vs. the private sector: Fifty-five percent of those in the public sector reported that they were responsible for 10 patients or less vs. 15% of those in the private sector. This is also reflected by the number of daily contacts: an average of 4.5 for psychologists in the public sector vs. 6.2 in the private sector.

### Patterns of treatment related to managed care

The professionals were asked to what extent (at their main employment) are the procedures of monitoring and control mandatory, what the type and duration of treatment are, how effectiveness is evaluated, and the extent of relations with primary physicians. In addition, to what extent did they take into consideration evidence-based treatment and financial/budgetary considerations? The patterns of treatment provision were examined only among professionals reporting direct therapy in their main employment (some 90% of all the professionals)^g^.

### Monitoring and control procedures

Among psychiatrists, 80% to 96% responded that – to a very large extent – they were obliged to document the diagnosis, to manage patient files regularly according to a uniform format, and to write up treatment summaries and recommendations vs. 50% of the psychologists who reported that they had to do this. Similarly, 70% to 80% of the psychiatrists responded that – to a large or very large extent – they were obliged to update the computerized patient files, document the cause of termination of treatment, set treatment goals, and document the treatment program; on the other hand, the rate of psychologists who so reported was 28% to 50%. The average score of the answers measuring the obligation to perform supervision and control procedures revealed that, to a large or very large extent, 63% of the psychiatrists complied with these procedures (an average score higher than 3.75) compared with 23% of the psychologists. At the same time, a difference was found in this average score between psychologists working mainly in the public vs. the private sector: 35% of the former scored higher than 3.75 as opposed to 14% of the latter.

In general, low rates of psychiatrists and psychologists reported an obligation to document periodic assessments of adherence to the treatment program and documentation of the outcome measures (16% to 25%); (Table 2).

**Table 2:** The extent supervision and control procedures should be performed (%)

**To what extent must you conduct the following procedures:**^**1**^	**Total**	**Psychiatrists**	**Psychologists**
Document the diagnosis**	67	96	55
Manage patient files in a uniform format**	60	86	49
Record a treatment summary and recommendations**	57	78	49
Document cause of termination of treatment**	55	68	50
Set treatment goals**	55	77	46
Document a treatment plan**	43	75	30
Update a patient’s computer file**	39	67	28
Document outcome measurement**	25	48	16
Document periodic assessment of adherence to treatment plan**	22	36	16
**Index of adherence to perform supervision and control procedures**^**2**^	35	63	23

N (population)=3,597.

n (sample size)=950.

^1^Rate of those responding “to a large extent” or “to a very large extent”.

^2^Rate of those scoring an average score over 3.7 on the 5-point scale from “not at all” to “to a very large extent”.

**The difference between the groups is significant, p<0.01.

### Type and duration of therapy (short- and long-term)

Most of the psychologists and psychiatrists, (some 95%), offer individual therapy. Sixty-one percent reported that they usually provide long-term treatment – for more than a year. Here, too, there was a significant difference between the professions. A higher rate of psychologists than psychiatrists (68% vs. 43% respectively) noted that most of their treatment was long-term. Concomitantly, a lower rate of psychologists than psychiatrists (9% vs. 34% respectively) noted that short-term treatment – up to half a year – characterized most of their treatment (Table 3).

**Table 3:** Distribution -duration of treatment, tools to assess effectiveness of treatment, evidence-based care and budgetary considerations (%)

	**Total**	**Psychiatrists**	**Psychologists**
Duration of treatment**	**100**	**100**	**100**
Short-term – up to half a year	16	34	9
Moderate – half a year to a year	23	23	23
Long-term – more than a year	61	43	68
**Tools to Assess Effectiveness of Treatment**^**1**^			
Therapist’s impression	92	92	92
Patients’ Self-reporting**	86	81	87
Reporting by other staff members**	39	60	31
Interviews**	26	56	14
Structured questionnaires**	14	26	10
Other	2	1	2
Non-use of tools	0.8	0.3	1
**Contact with primary physician**^**2**^	54	85	42
**Updated on evidence-based treatment**^3^**	32	65	20
**Evidence-based care is a consideration in the choice of treatment plan**^3^**	25	57	13
**Budgetary considerations**^3^*	32	37	30

N (population)=3,597.

n (sample size)=950.

**The difference between the professions is significant p< 0.01.

*The difference between the professions is significant p< 0.05

^1^The figures do not add up to 100% as more than one response was possible.

^2^Rate reporting contact with primary physicians.

^3^Rate responding “to a large extent” or “to a very large extent”.

The difference in duration of therapy was also connected to the private vs. the public sector and to community vs. hospital settings: Multivariate analysis (logistic regression) revealed that psychologists and psychiatrists working in the community and the private sector are more likely (thrice and twice as much respectively) to provide long-term treatment (of more than a year) than therapists in the public sector or in a hospital (see Table 4).

**Table 4:** Multivariate analyses – logistic regression

**Variables predicting long-term treatment (n=950)**				
**Variable**	**Reference**	**Regression coefficient (B)**	**Odds-ratio**	**Confidence interval**
20- years of experience	20+ years of experience	0.2	1.2	1.7-0.9
Work in the community*	Hospital work	1.2	3.4	5.7-2.1
Psychologist	Psychiatrist	0.1	1.2	1.8-0.8
Private sector	Public sector	0.8	2.3	3.2-1.6
Trained in Israel	Trained abroad	0.2	1.2	1.8-0.8
Women	Men	0.3	1.4	1.9-1.0
Constant*		−1.5	0.2	1.7-0.9
**Variables Predicting Use of Evidence-Based Care in Planning Treatment (n=1030)**				
20- years of experience	20+ years of experience	0.3	1.4	2.0-0.9
Work in the community	Hospital work	−0.4	0.7	1.2-0.4
Psychiatrist**	Psychologist	1.9	6.9	10.8-4.4
Public sector*	Private sector	0.5	1.7	2.6-1.1
Trained in Israel	Trained abroad	0.2	1.2	1.9-0.7
Men**	Women	0.5	1.7	2.5-1.2
Constant**		−2.3	0.1	
**Variables Predicting Financial Considerations (n=1,030)**				
20- years of experience	20+ years of experience	0.1	1.1	1.5-0.8
Work in the community	Hospital work	0.2	1.2	2.0-0.8
Psychiatrist	Psychologist	0.2	1.3	1.9-0.8
Public sector*	Private sector	0.4	1.4	2.0-1.0
Trained in Israel	Trained abroad	0.1	1.1	1.6-0.7
Men**	Women	0.5	1.6	2.3-1.2
Constant**		−1.5	0.2	

*p<0.05.

**p<0.01.

### Tools to measure effectiveness of therapy

When asked which tools they used to assess the effectiveness of their treatment, the professionals cited more than one (more than one answer could be given). One tool used by most (92%) was personal impression. In addition, 86% used self-reporting by patients; 60% of the psychiatrists and 31% of the psychologists relied on reporting by other staff members; some 56% of the psychiatrists and 14% of the psychologists used interviews; and some 26% of the psychiatrists and 10% of the psychologists used structured questionnaires. Very few answered that they used no tools to assess the effectiveness of treatment (Table 3).

### Contact with the primary-care physicians

Of the psychiatrists, 85% said that they had contact with their patients’ primary care physicians in one or more of these respects: they informed physicians of the fact of treatment, referred patients to physicians for medication (or the physicians established the initial contact and referred patients to them), medically advised physicians at primary clinics (liaison), held joint meetings.

About 40% of the psychologists reported some contact with primary-care physicians, mainly in the form of referrals for medication (or the physicians contacted and referred patients to them – Table 3).

In the multivariate analysis (logistic regression), the following, too, entered the equation as independent variables: Community work, public-sector work, place of training, number of years in the profession, and gender. It was found that psychiatrists were 10 times as likely as psychologists of maintaining contact with the primary-care physicians. Moreover, the likelihood of maintaining contact with primary-care physicians was greater in the community and public sector than in hospitals or the private sector (this finding does not appear in the table).

### Evidence-based treatment and budgetary/financial considerations

A high rate of psychiatrists responded that they were up-to-date on evidence-based care to a large or very large extent (65%), and that the provision of this type of care was a consideration in the choice of a treatment plan at their main place of work (57%). In comparison, 20% and 13% respectively of the psychologists gave this response (Table 2). Moreover, multivariate analysis (logistic regression) revealed that the likelihood of psychiatrists reporting that they took into account evidence-based care was seven times that of psychologists. In addition, for psychiatrists and psychologists in the public sector, and for males, the likelihood of giving this response were twice that of those working in the private sector, and of women (Table 4).

About a third of all the professionals reported that they take financial/budgetary considerations into account to a large extent (Table 3). In the public sector, the odds of giving this response were somewhat greater (1.4 times) than in the private sector. Similarly, the likelihood of men reporting this was 1.6 times that of women.

### Perceived impact of the insurance reform in mental health

Table 5 presents the rate of psychologists and psychiatrists who, to a large or very large extent, expect the reform to change their working arrangements and the provision of care. Of the former, 70% compared with 50% of the psychiatrists expect change in the provision of care (with the emphasis on short-term methods, shorter duration of therapy, prominence of medication, less psycho-dynamic testing, and less outreach); concomitantly, 59% of the psychologists vs. 35% of the psychiatrists expect change in the quality of care (unsuitable or insufficient therapy, detriment to the severely mentally ill, use of unsuitable service providers, and more referral to groupwork); similarly, 52% vs. 39% of the psychiatrists expect change in their work patterns (more consideration of financials, increased use of forms, paperwork and bureaucracy, supervision of diagnoses and interference in professional considerations, emphasis on direct contact with patients, and reduction of inter-services care). Fifty percent of the psychologists vs. 34% of the psychiatrists expect change in professional training (reduction of staff training/instruction, fewer hours of consultation and staff meetings, less time devoted to professional updating and courses, reduction of scope and range of professional training areas); and 48% of the psychologists vs. 24% of the psychiatrists expect change in the employment market (preference for “less expensive” therapists, detriment to workers’ rights and income). One exception is the finding that a high rate of professionals in both groups, psychologists and psychiatrists, do not expect substantial change in the type and number of patients subsequent to reform (Table 5).

**Table 5:** Rate of respondents who “to a large extent” expect changes in their working patterns and the provision of care (%)

**Measure of change in:**	**Psychiatrists**			**Psychologists**		
	**Respondents’ rate**^**1**^	**Mean**	**Std.**	**Respondents’ rate**^**1**^	**Mean**	**Std.**
Treatment process Index**	50	3.64	0.79	70	3.98	0.67
Patterns of work Index**	39	3.53	0.78	52	3.75	0.72
Quality of care Index**	35	3.35	0.82	59	3.81	0.70
Training and specialization Index**	34	3.29	1.10	50	3.75	0.94
Employment market Index**	24	3.27	0.90	48	3.73	0.87
Type and number of patients Index**	16	2.72	0.92	7	2.58	0.76

**The difference between the groups is significant; p<0.01.

^1^The rate of those scoring higher than 3.75 on a scale of 1 to 5, from “not at all” to “to a very large extent”. 3.75 was chosen as the cut-off point between high and low in the responses on the measure in order to include those who answered on most (but not all) items either 4 (“to a large extent”) or 5 (“to a very large extent”).

A higher rate of psychologists in the public sector expect their work patterns to change with respect to the treatment processes of training and specialization (60%, 74% and 55% respectively) compared with their colleagues in the private sector (45%, 66% and 44% respectively).

When asked which changes they expect in professional standards, half answered that there would be emphasis on defining and meeting the treatment goals. A third believed that there would be more transparency; similarly, a third felt that confidentiality would suffer. At the same time, only a third thought that the reform would improve availability and accessibility of care.

In multivariate analyses (logistic regression), it was found that professionals offering treatment of medium duration on average expected the employment market change to a somewhat smaller extent than those offering long-term treatment. The former compared with the latter also expected the reform to change to smaller extent the quality of care. Professionals reporting that they already offered evidence-based care or took financial considerations into account expected the quality of care to change to a smaller extent than others – though the significance was border-line. In addition, women and professionals who were trained in Israel expected the reform to change to a greater extent the employment market and the quality of care (Table 6).

**Table 6:** Variables impacting the extent of change expected (linear regression analyses)

**Variables impacting the extent of change expected in the employment market**			
**Variable**	**B**	**β**	**Std. error**
Psychologist**	0.31	0.15	0.10
Women*	0.18	0.09	0.08
**Working in public sector**	0.07	0.04	0.08
**Duration of treatment**			
Short-term – up to half a year	-0.09	-0.04	0.10
Moderate – half a year to a year*	-0.17	-0.08	0.09
**Evidence-based care**	-0.04	-0.02	0.09
**financial considerations**	-0.10	-0.05	0.08
Trained in Israel**	0.29	0.14	0.09
**R**^**2 **^**- 10%**			
**Variables Impacting the Extent of Change Expected in the Quality of Care**			
Psychologist**	0.29	0.17	0.08
Women**	0.22	0.13	0.06
Working in public sector*	0.16	0.10	0.06
**Duration of treatment**			
Short-term – up to half a year	-0.11	-0.05	0.08
Moderate – half a year to a year*	-0.17	-0.09	0.07
**Evidence-based care**^**†**^	-0.13	-0.07	0.08
**financial considerations**^**†**^	-0.12	-0.07	0.06
Trained in Israel**	0.24	0.13	0.07
**R**^**2 **^**- 13%**			

*p<0.05.

**p<0.01.

^†^p<0.1 (borderline significance).

Note: reference for psychologists – psychiatrists; reference for women – men; reference for working in the public sector – working in the private sector; reference for short-term – up to half a year, moderate term – half a year to a year, long-term – more than a year; reference for evidence-based care – those not reporting thus; reference for allowing for financial considerations – those not reporting thus; reference for studies in Israel – studies abroad.

## Discussion

Although psychiatrists and psychologists treat a population with a similar range of problems (albeit of a different mix), substantial and statistically significant differences were found in their personal and professional background characteristics, their work patterns and treatment provision, and their expectations regarding the effect of reform. Some of these differences were connected to the differences between working in the private sector (applicable to a high rate of psychologists) and the public sector.

The study identified gaps between the treatment provision of some professionals, mostly psychologists, and the demands of a managed-care environment. A high percentage of professionals reported offering long-term treatment whereas managed-care organizations encourage short-term care [11]. A low percentage of psychologists reported compliance with monitoring and control procedures whereas managed-care organizations demand compliance as a way to curb costs and ensure quality [5,6]. A substantial rate of psychiatrists reported a high level of updated knowledge in the field of evidence-based care, the provision of which was a consideration in their choice of a treatment program at their main place of work. Far lower rates of psychologists responded thus.

In addition, a low rate of professionals reported using structured tools to measure the effectiveness of treatment. This is expected to change with the transfer to the health plans: in the transition to managed care in the US, the use of outcome measurement rose along with the demand to demonstrate the effectiveness of psychological intervention [12,19-21]. In addition, some 60% of the psychologists had no contact with primary-care physicians about their patients (vs. 15% of the psychiatrists). The transfer of mental-health services to the health plans is expected to better integrate mental and physical health and to change the relationship between therapists and primary-care physicians [22,23]. One partial change in Israel in recent years is that primary care physicians already do regard themselves as the community resource person to turn to in mental distress [24]. It should be pointed out that once the mental health insurance reform goes into effect, health plans will be offering wide geographic access to free or very low-cost mental health care. They are expected to provide this care through a mix of health plan-owned clinics and independent clinics. It is likely that the demand for mental health services provided by the health plans will increase (which might or might not lead to a decrease in demand for private services). Accordingly, the health plans will need to recruit and work with a growing number of practitioners. In a previous study [3] it was found that the health plans are planning to increase the number of psychologists, psychiatrists and social workers with whom they work. Some of these are likely to be drawn from the private sector, while others are likely to be drawn from the governmental services. Some of today's private sector practitioners may ultimately decide to work with the health plans, while others may choose to stay with private work only.

## Conclusions

• The findings reveal that psychiatrists and psychologists with experience in aspects of managed care – evidence-based care, consideration of financials, moderate-term therapy – have less expectation than others of negative, post-reform changes in the quality of care and the employment market. Apparently, their need for an adjustment process to the health plans will be smaller than the others’.

• At the same time, a high rate of psychologists – more than half, working mainly in the private sector – reported that they had no evidence-based knowledge, that it did not constitute a consideration in their choice of a treatment plan, and that they had no contact with their patients’ primary-care physicians. More so than the psychiatrists, they expected (negative) changes in the provision of care, quality of care, training, and the employment market. Also, a higher rate of them did not expect improvement in the accessibility and availability of services following reform. They, apparently, will require more adjustment to working with the health plans.

• Consequently, according to the arrangements being worked out with the health plans, they will be able to deliver services via private practitioners as well, beyond the clinic framework; i.e., they might wish to recruit a large number of therapists from the private market. it seems that the efforts to incorporate methods and approaches suitable to treatment in an era of managed care should focus on this group of professionals. To cope with these challenges, it is important to pay attention to the various differences between psychologists working mainly in the public sector and those in the private sector.

• In mobilizing for the reform, the health plans must prepare to recruit many professionals unaccustomed to working in managed-care frameworks. This means they should construct tracks to lead experienced, skilled specialists to the public system. In this connection, the health plans could offer the professionals various models of joint work and training for the new environment (e.g., the required mechanisms of monitoring and control and, the preferred methods of treatment). It is also important for the health plans to prepare, by learning the language and thinking of the professionals, since, to a large extent, the encounter will be between two different organizational cultures.

• Preferably, the adaptation processes should start during the periods of training and specialization. These might include topics related to clinic management, methods of measuring effectiveness of treatment, and even forms of cooperation between mental-health professionals, including primary physicians.

Working intensively with professionals in mental health, particularly with psychologists, is a relatively new experience for the health plans. The research findings may well contribute to a better understanding of the professions and their approaches to treatment, improving communication with the service providers.

This is a preliminary, cross-sectional study at a specific point in time. It is designed to describe the work of core professionals in mental health in Israel on the eve of imminent reform in order to establish a baseline for a future study on the implications of the reform. As such, it is restricted to a description of the situation at this point in time. In future studies, it would be important to examine the implications over time, with the emphasis on the various impacts on professionals in the public vs. the private sectors including the impact on the professional specialization process. Similarly, it would be important to examine the impact on other professionals delivering services in mental health, such as social workers, occupational therapists and nurses.

## Endnotes

^a^Social workers employed in the field of mental health were also included in the study. However, there is no formal licensing in Israel for mental-health social workers and, therefore, no orderly listings of those employed in this field. Thus, we did not have a defined sampling framework and could not construct a representative sample of this population. The study questionnaire was sent out to 283 social workers employed in Ministry of Health frameworks. Due to the differences in sampling and data collection, the results were analyzed separately from those for psychologists and psychiatrists. The results may be accessed in the research report on the Brookdale website: http://www.jdc.org.il/brookdale.

^b^Main place of employment (main job) is where one works for most of the week or – if one works at similar jobs in different places – that which is considered the main employment.

^c^The formulation of the components of each measure may be found, in Hebrew, in Appendix III of the research report: http://www.jdc.org.il/brookdale.

^d^Overall, 52 percent of the psychologists and 88 percent of the psychiatrists work for the public sector (either primary or secondary employment; either full time or part time).

^e^The study findings showed that, as their main employment, the vast majority (87%) of psychologists work in the community (anywhere outside the hospital, either in public clinics or in private clinics), whereas more than half of the psychiatrists (52%) work in hospitals. Note, however, that hospital work includes service delivery in ambulatory clinics which serve community patients, and in community clinics located on hospital grounds. To be sure, an examination of the type of patients treated found that more psychiatrists than psychologists provided care in cases of “hard” psychiatry. At the same time, significant overlap was found in cases of “soft” psychiatry. Similar rates of psychiatrists and psychologists reported treating mainly anxiety disorders, personality disorders, and trauma (i.e., these disorders comprise more than 10% of the conditions they treat) with no significant difference between the two professions. Other disorders of “soft” psychiatry (which make up most of the volume of community mental health), such as life crises and depression were reported at high rates by both professions though more significantly by psychologists than psychiatrists (70% vs. 50% and 68% vs. 53% respectively).

^f^The question was: “In the course of treatment, what percentage of your patients receive an average of: one session per week, 2–6 sessions, 7–12 sessions…”

^g^Note, that the question asked whether the respondent was *required* to undertake these practices. It may be that some respondents undertake these practices, without being required.

## Abbreviations

NHIL: National health insurance law; MoH: Ministry of Health; MBHOs: Managed Behavioral Health Organizations.

## Competing interests

The authors declare they have no competing interests.

## Authors’ contributions

NN initiated the study and was the leading researcher, responsible for the design, questionnaire construction, oversight of fieldwork and data analysis, and drafting the manuscript. HS contributed to the study design and questionnaire construction, the fieldwork and data analysis. Both authors contributed to the writing of the manuscript, and read and approved the final draft.

## Authors’ information

NN is a senior researcher at the Smokler Center for Health Policy Research of the Myers- JDC-Brookdale Institute. She has an M.A. degree in Labor Studies from Tel Aviv University.

HS is a researcher at the Smokler Center for Health Policy Research of the Myers- JDC-Brookdale Institute. She has an M.A. degree in Public Policy from the Hebrew University of Jerusalem.
